# Tools to tipple: ethanol ingestion by wild chimpanzees using leaf-sponges

**DOI:** 10.1098/rsos.150150

**Published:** 2015-06-09

**Authors:** Kimberley J. Hockings, Nicola Bryson-Morrison, Susana Carvalho, Michiko Fujisawa, Tatyana Humle, William C. McGrew, Miho Nakamura, Gaku Ohashi, Yumi Yamanashi, Gen Yamakoshi, Tetsuro Matsuzawa

**Affiliations:** 1 Centre for Research in Anthropology (CRIA-FCSH/UNL), Lisbon 1069-061, Portugal; 2 Anthropology Centre for Conservation, Environment and Development, Oxford Brookes University, Oxford OX3 0BP, UK; 3 School of Anthropology and Conservation, University of Kent, Canterbury CT2 7NR, UK; 4 Center for the Advanced Study of Hominid Paleobiology, George Washington University, Washington DC, 20052, USA; 5 Interdisciplinary Center for Archaeology and Evolution of Human Behavior, Universidade do Algarve, Faro 8005-139, Portugal; 6 Primate Research Institute, Kyoto University, Inuyama 484-8506, Japan; 7 Center for Southeast Asian Studies, Kyoto University, Kyoto 606-8501, Japan; 8 Department of Archaeology and Anthropology, University of Cambridge, Cambridge CB2 1QH, UK; 9 Wildlife Research Center, Kyoto University, Kyoto 606-8203, Japan; 10 Japan Monkey Centre, Inuyama 484-0081, Japan; 11 Chubu University, Kasugai 487-8501, Japan; 12 Center for African Area Studies, Kyoto University, Kyoto 606-8501, Japan

**Keywords:** ethanol ingestion, elementary tool-use, great apes, raffia palm

## Abstract

African apes and humans share a genetic mutation that enables them to effectively metabolize ethanol. However, voluntary ethanol consumption in this evolutionary radiation is documented only in modern humans. Here, we report evidence of the long-term and recurrent ingestion of ethanol from the raffia palm (*Raphia hookeri,* Arecaceae) by wild chimpanzees (*Pan troglodytes verus*) at Bossou in Guinea, West Africa, from 1995 to 2012. Chimpanzees at Bossou ingest this alcoholic beverage, often in large quantities, despite an average presence of ethanol of 3.1% alcohol by volume (ABV) and up to 6.9% ABV. Local people tap raffia palms and the sap collects in plastic containers, and chimpanzees use elementary technology—a leafy tool—to obtain this fermenting sap. These data show that ethanol does not act as a deterrent to feeding in this community of wild apes, supporting the idea that the last common ancestor of living African apes and modern humans was not averse to ingesting foods containing ethanol.

## 1. Background

The ‘drunken monkey hypothesis’ states that natural selection favoured those primates with an attraction to ethanol (commonly referred to as alcohol) because it was associated with proximate benefits (e.g. acting as an appetite stimulant or a cue to finding fruit, or as an unavoidable consequence of a frugivorous diet, etc.), consequently increasing caloric gains [1,2]. Anecdotes about wild non-human primates ingesting ethanol abound, although most remain non-validated, with few exceptions. The slow loris (*Nycticebus coucang*) ingests fermented nectar (of up to 3.8% ethanol content) from the Bertam palm (*Eugeissona tristis*) [3]. Introduced green monkeys, *Chlorocebus sabaeus*, on St Kitts target tourist cocktails, although quantitative behavioural data examining ethanol consumption have been published only on captive monkeys in a nearby facility [4,5]. Empirical data on primate ethanol ingestion in nature are required to examine the conditions that might favour ethanol consumption in primates, for example as a by-product of frugivory [6]. However, Milton [7] argued based on questionnaires on primate feeding behaviour directed at field primatologists that primates are not attracted to, and rarely eat, over-ripe fruit (which contain higher levels of ethanol than unripe or ripe fruit [8]), and so intoxication in the wild is almost non-existent.

Despite the apparent rarity of ethanol ingestion among non-human primates in nature, ethanol consumption occurs in every modern human society that has access to fermentable raw materials [9]. Hence, it has been suggested that ethanol ingestion provided an adaptive benefit to human ancestors, and by extension, perhaps also other apes. From different points in the approximately 70 Myr of primate evolution, Carrigan *et al.* [10] resurrected ancestral alcohol dehydrogenase 4 enzymes (an enzyme expressed in the upper gastrointestinal tract) and identified a single mutation occurring about 10 Ma, when the last common ancestor of living African apes and modern humans [11] experienced a genetic mutation that increased by 40-fold the rate that ethanol was metabolized. Our observational study details habitual (i.e. occurs repeatedly in several individuals) ethanol consumption among Bossou chimpanzees in West Africa, thus bolstering this molecular finding by demonstrating that ethanol does not act as a deterrent to feeding in this community of wild apes.

## 2. Methods

Raffia palms, which occur naturally in seasonally flooded areas [12], are traditionally tapped by humans close to the crown of mature palms, which produces fermented palm sap year-round [13]. The natural sugars in the palm sap quickly ferment into ethanol. Villagers at Bossou, Guinea install a modified plastic container (of 5–30 l volume) to collect the dripping sap, and then cover the container with leaves to avoid contamination. The tappers collect all the fermented sap from the container in the early morning (06.00–08.00 h) and late afternoon (16.00–18.00 h), and it is usually consumed quickly (i.e. within 24 h) without processing.

Local harvesters are fully aware of the increased risks of tapping raffia palms within the chimpanzees' home range area, but do not remain in proximity to the palm throughout the day, making it difficult to guard against chimpanzee feeding. Over the years, Bossou researchers have witnessed chimpanzees ingesting the palm sap; several individuals sometimes take part in a single drinking session. Each individual was coded for only one event per session (for independence of data-points). We defined a drinking *event* from when an individual inserted a drinking tool into the container until that individual ceased drinking and descended to the ground. We defined a drinking *session* from when the first individual of a party started to drink until the last remaining chimpanzee at the top of the crown ceased drinking and descended the palm. Within a drinking session, we analysed drinking events per individual. Individuals of 12 years and older were classified as adults, and individuals aged less than 12 years were classed as immatures [14]. Infants (0–3 years) were excluded from all analyses, as they did not make and use their own drinking tools [15]. The duration of drinking events was included only if we saw the complete drinking event. We did not test individual differences in palm sap ingestion, as the number of observations per individual was too low.

Chimpanzees use leaves as tools in various contexts. To ingest fluids, chimpanzees often use leafy tools, a technique that is ubiquitous among wild chimpanzee communities [16]. An individual detaches leaves, and then folds or crumples them inside the mouth to produce a drinking tool, i.e. a leaf-fold, leaf-scoop or a leaf-sponge [17,18]. The tool is immersed in the fluid, then retrieved and inserted into the mouth for compressive extraction between the tongue and palate [19]. We analysed 12 drinking events from video-clips (from seven individuals FF, YL, JJ, VI, Yo, Jr, Ju, over six sessions) in which the drinking individual was clearly observable, to calculate the rate of drinking (i.e. how many dips per min). Adults averaged 9.25 dips min^−1^ (*n*=9, s.d.: 2.21) and immatures averaged 9.7 dips min^−1^ (*n*=3, s.d.: 4.40). Tonooka [17] calculated that in each drinking action, an individual (age-classes combined) sucked-up approximately 10 ml of water using a leaf tool, but the range was probably 10–50 ml per dip [15,19]. For calculations, we use the 10 ml value as a conservative measure of fluid obtained per dip. However, as leafy tools made by immatures were less efficient than those of adults (tended to be smaller and carry a smaller volume of water) [15,18,19], we used data only from adult individuals for subsequent analyses. We estimated how many dips an adult individual made based on the duration of the drinking event (i.e. drinking duration in minutes multiplied by 9.25).

**Figure 1.**
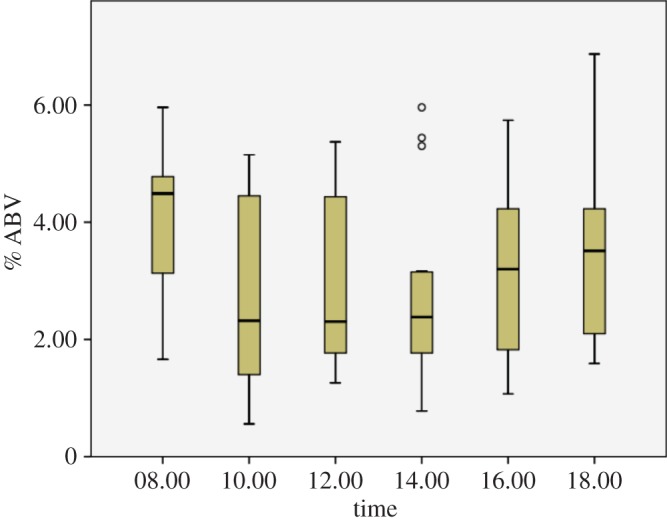
Box plot (showing maximum and minimum values as whiskers above and below the central rectangle which spans the first quartile to the third quartile; the segment inside the rectangle shows the median, and outliers are open circles) for % alcohol by volume (ABV) of palm sap from 16 raffia palms, collected at 2 h intervals (i.e. 08.00–18.00 h) throughout the day. Data presented in figure 1 come from 88 samples (maximum of six samples collected daily per raffia palm; data were unavailable for only eight of 96 samples that could have been collected, see electronic supplementary material, table S1). Sap sampled at 08.00 h had accumulated overnight, about 14 h after the previous batch of fermented sap was harvested. Following the morning harvest, fresh sap accumulated in the container until the evening harvest.

We calculated the ethanol content (% alcohol by volume (% ABV)) of palm sap using refractometer (Zeiss) and hydrometer (SG) readings from 16 raffia trees occurring within the chimpanzees' home range. We collected samples at times when chimpanzees usually obtained the accumulated sap. Typical accuracy of these instruments is generally ±0.5% ABV. We took all measurements within the temperature limits of the refractometer and avoided direct sunlight. For each sample, 100 ml of palm sap was collected *in situ* at the morning harvest (06.00–08.00 h), and every 2 h thereafter, until the evening harvest (16.00–18.00 h), and hydrometer and refractometer readings were taken immediately. To ensure that calculations of ethanol quantities ingested by chimpanzees represented the time of day a chimpanzee drinking session occurred, we divided time of day into two categories: (i) high ethanol—08.00, 16.00, 18.00 h, where sap averaged more than 3% ethanol, and (ii) low ethanol—10.00–14.00 h where sap averaged below 3% (figure 1). During the high ethanol time of day, sap averaged 3.5% ABV. During the low ethanol time of day, sap averaged 2.8% ABV. For each adult fermented palm sap drinking event for which drinking duration data were available (*n*=28), we calculated the approximate amount of ethanol ingested by each individual (ml). Means are presented with standard errors.

## 3. Results

The ethanol content of palm sap collected from 16 raffia palms within the Bossou chimpanzees' home range averaged 3.1±0.2% ABV. Between 06.00 and 18.00 h, the maximum ethanol content recorded was 6.9% ABV (figure 1). The elapsed time since the sap in the container had last been collected influenced the ethanol content of the sap (i.e. the ethanol content of sap draining into the container continued to increase after the early morning or late afternoon collection; electronic supplementary material, table S1), but trees also varied significantly (repeated-measures ANOVA: time: *F*_5,67_=5.42, *p*<0.001; tree: *F*_15,67_=18.16, *p*<0.001).

**Figure 2.**
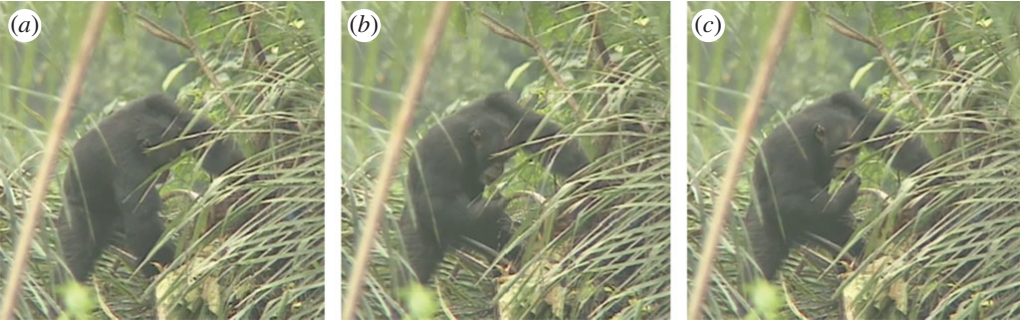
Adult male chimpanzee (FF) uses a leaf tool to drink raffia sap from a container (see also electronic supplementary material, video S1): (*a*) FF inserts right hand holding the leaf tool into the fermented palm sap container, (*b*) retrieves the leaf tool that is soaked in fermented palm sap and (*c*) transfers the soaked leaf tool to his mouth to drink the palm sap it carries (photos by M. Nakamura, 28 December 1996, see electronic supplementary material, table S2, session 2).

Over 17 years, we observed 51 fermented palm sap drinking events recorded during 20 drinking sessions involving 13 adult and immature individuals (electronic supplementary material, table S2). Chimpanzees always used a leaf tool to drink, making a crushed or folded leaf ‘sponge’ from the leaves placed as a protective covering by villagers or from plants growing nearby. This absorbent extractive tool was dipped into the small opening of the fermented palm sap container (see [19] for an ethogram of leaf tool-use), then retrieved and put into the mouth for drinking. Individuals either co-drank, with drinkers alternating dips of their leaf-sponges into the fermented palm sap [20], or one individual monopolized the container (figure 2 and electronic supplementary video S1, also see [21]), while others waited their turn. Thirteen of 26 adult and immature individuals present in the Bossou community between 1995 and 2012 (excluding infants) were never seen ingesting palm sap (see [21] for Bossou lineage).

Chimpanzees drank palm sap at a rate of 0.18 sessions 100 *h*^−1^ (i.e. three drinking sessions were observed in 1673 h of observation over a 12-month period) [22]. Drinking sessions occurred across months and seasons and at various times of the day (from 07.00 to 17.54 h), with no evidence of chimpanzees ingesting palm sap at night. Adult individuals averaged 9.25±0.74 dips per min (calculated from nine video-clips of independent drinking events). Adult drinking events ranged from 50 to 1920 s (mean: 636 *s*±103, *n*=28 events, six individuals), with individuals averaging 1.0±0.2 *l* of fermented palm sap per drinking event (*n*=28; *range*=0.1–3.0 l). Males accounted for 34 of the 51 events; however, one adult male in particular (FF) accounted for 14 of 51 events (electronic supplementary material, table S2). The amount of ethanol ingested per event ranged from about 2.5 to 84.9 ml (electronic supplementary material, table S2), with no sex difference (males: *mean*=31.3±5.3; females: *mean*=25.1±11.8; one-way ANOVA: *F*_1,26_=0.319, *p*=0.57). An individual that ingested a small quantity of palm sap in one drinking event could ingest a greater quantity in other events, and an individual that ingested a high quantity in one event was not averse to ingesting ethanol again shortly thereafter (electronic supplementary material, table S2).

## 4. Discussion

We document the first quantitative assessment of chimpanzees ingesting ethanol in the wild. Chimpanzees ingest fermented palm sap at Bossou rarely, but habitually. They use leaves to soak up and drink the sap, and like other chimpanzee communities that produce tools for drinking (e.g. moss-sponging at Budongo, Uganda [23]), the behaviour is consistent with some degree of social transmission [16]. All age and sex classes ingested palm sap, and there was no sex bias in the quantity of ethanol ingested during a feeding event. In addition, there does not appear to be a pattern in the amounts ingested by the same individual in different events. Some of the chimpanzees at Bossou consumed significant quantities of ethanol and displayed behavioural signs of inebriation. Researchers rarely collected detailed behavioural data before versus after exposure to ethanol, but some drinkers rested directly after imbibing fermented sap.

Fruits ferment naturally and regular ethanol consumption in the wild may be an unavoidable consequence of a frugivorous lifestyle. In the cases documented here, chimpanzees drank the fermented sap of raffia, and hence ethanol consumption was not a direct by-product of frugivory. Furthermore, raffia palm sap drinking is opportunistic, relying on a person having installed the specialized equipment to drain the sap from a mature palm. Other factors beyond the apes' control limit its exposure to fermented palm sap: raffia palms are scarce within the chimpanzees' core range (being mostly found in wetter areas of their range), and while a palm may produce fermented sap for several weeks, it dies after production.

Raffia sap has a rich composition of vitamins and minerals [24], and provides energy in the form of sugars, mostly sucrose and glucose. The simple sugar content [25], approximately 10%, makes it sweet and palatable, even with ethanol. The raffia palm sap ferments very quickly and the ethanol produces a distinctive odour and taste. Nonetheless, we observed individuals repeatedly consuming fermented palm sap—often in large quantities—suggesting that accidental ethanol ingestion is unlikely. Our results clearly indicate that ethanol is not an absolute deterrent to chimpanzee feeding in this community. An experimental trial to provide chimpanzees with access to fermenting and non-fermenting palm sap is needed to test if gustation is a straightforward proximate explanation for the fermented palm sap ingestion (i.e. whether ethanol is an attractant).

Chimpanzees are intelligent, highly dextrous and variably technical; they use tools to access hard-to-reach food sources [26,27]. Lacking natural containers, Bossou chimpanzees use leaf tools to access fermented palm sap that has been tapped by people, and show no evidence of being able to obtain sap without human affordance. However, wild sources of ethanol (including fermented fruits and nectars) are available to chimpanzees, as for other primates [2,3], but quantitative measurements of ethanol coupled with direct observations of feeding behaviour are as yet lacking. Unlike examples of primates ingesting anthropogenic sources of ethanol elsewhere, such as introduced green monkeys at St Kitts targeting tourist cocktails, chimpanzee attraction to fermented palm sap at Bossou does not result from former provisioning of ethanol by local people.

Carrigan *et al.* [10] suggest that an enhanced ability to metabolize ethanol in the last common ancestor of living African apes and humans may have arisen from a more terrestrial lifestyle with its increased likelihood of encountering fermenting fruits on the ground. Apes rarely feed on fleshy fruits after they have fallen to the ground, although some fruit species, such as *Parinari* spp. and *Treculia africana*, are often gathered by chimpanzees from the ground and eaten [28–30]. Data on ethanol ingestion (if present) among gorillas (*Gorilla gorilla*) and bonobos (*Pan paniscus*) would be useful to understand the conditions that favoured this molecular adaptation. Further palaeogenetic and ethological research may offer insight into potential subspecies and interpopulation differences in chimpanzees' ability to oxidize ethanol [10]. Relevant are the roles of different alcohol dehydrogenases [31], the ethanol content of different fruits that vary in their defences against fermentation, and chimpanzee strategies when foraging on foods containing ethanol (i.e. their importance in the diet or role as fallback foods). Examination of these factors should allow further specific testing of the ‘drunken monkey hypothesis’.

## Supplementary Material

Table S1. The % ABV of palm sap from 16 raffia palms, collected at 2-hr intervals (i.e. 08:00-18.00 h) throughout the day. Table S2. Palm wine drinking sessions and events by chimpanzees, including date, start time, chimpanzee name, age (yr) and sex of imbiber, palm wine drinking duration (min), quantity of palm wine consumed (l), leaf dip rate (dips per min, calculated from video clips), estimated amount ethanol ingested (ml), and initials of researcher who contributed data. Video S1. Video clip of an adult male chimpanzee, Foaf, drinking fermented palm sap using a leaf tool.

## Data accessibility

Data can be found in the electronic supplementary material.
