# Supplementary material for: Tools to tipple: ethanol ingestion by wild chimpanzees using leaf-sponges
Source: R Soc Open Sci. 2015 Jun 9;2(6):150150. doi: 10.1098/rsos.150150 (PMC4632552; doi:10.1098/rsos.150150)
Supplement: Table S1. The % ABV of palm sap from 16 raffia palms, collected at 2-hr intervals (i.e. 08:00-18.00 h) throughout the day. Table S2. Palm wine drinking sessions and events by chimpanzees, including date, start time, chimpanzee name, age (yr) and sex of imbiber, palm wine drinking duration (min), qua [file rsos150150supp1.docx]

**Electronic Supplementary Material**

Table S1. The % ABV of palm sap from 16 raffia palms (rounded to 1 decimal place), collected at 2-hr intervals throughout the day (i.e. 08:00-18.00 h). Data come from 88 samples (maximum of 6 samples collected daily per raffia palm; data were unavailable for only 8 of 96 samples that could have been collected due to insufficient sap production for testing). Sap sampled at 08:00 h had accumulated overnight, about 14 h after the previous batch of fermented sap was harvested. Our sample collection methods might have underestimated the ethanol content in palm sap: the removal of 100ml of palm sap every 2 h from a raffia palm producing a small quantity of sap meant that the newly produced sap (that had not yet fermented) would not mix with existing sap in the container (that had already started fermentation). The latter might explain why the %ABV of sap did not always increase steadily from 10:00 h to 18:00 h. Although untested, various factors might influence the % ABV of palm sap, including the location of the tree (i.e. in drier or wetter parts of the habitat) and the amount of light a tree receives, the time since the tree was tapped and the amount of sap each tree has already produced, local people’s individual tapping and harvesting practices, including the particular container used to collect sap and the amount of sap left in the vessel after each harvest, and variation in compounds produced by the tree or introduced by humans.

|  | **% ABV** | | | | | |
| --- | --- | --- | --- | --- | --- | --- |
| **Tree** | **Time** | | | | | |
|  | **08:00** | **10:00** | **12:00** | **14:00** | **16:00** | **18:00** |
| **1** | 1.7 | 1.2 | 1.3 | 1.6 | 1.5 | 1.6 |
| **2** | 2.3 | 1.7 | 2.5 | 2.6 | 3.4 | 3.5 |
| **3** | 4.8 | 3.5 | 4.0 | 2.9 | 1.7 |  |
| **4** | 4.8 | 4.7 | 4.8 | 2.2 | 4.0 |  |
| **5** | 2.7 | 1.4 | 1.8 | 3.2 | 4.0 | 3.5 |
| **6** |  |  | 2.2 | 2.2 | 2.4 | 2.5 |
| **7** | 3.1 | 1.3 | 3.5 | 3.1 | 3.9 | 3.6 |
| **8** | 5.5 | 5.2 | 4.9 | 5.3 | 5.4 | 5.4 |
| **9** | 6.0 | 4.7 | 5.3 | 6.0 | 5.7 | 6.9 |
| **10** | 5.5 | 4.5 | 5.4 | 5.4 | 4.5 | 4.2 |
| **11** | 3.2 | 2.3 | 2.4 | 2.6 | 4.5 | 4.3 |
| **12** |  |  | 1.4 | 0.8 | 1.1 | 2.3 |
| **13** |  |  | 1.8 | 1.2 | 2.0 | 2.1 |
| **14** | 4.6 | 2.4 | 1.3 | 1.5 | 1.2 | 1.9 |
| **15** | 4.5 | 2.2 | 2.0 | 2.0 | 3.0 | 3.5 |
| **16** | 3.4 | 0.6 | 1.9 | 1.9 | 2.0 | 2.1 |

Table S2. Palm wine drinking sessions and events by chimpanzees, including date, start time, chimpanzee name, age (yr) and sex of imbiber, palm wine drinking duration (min), quantity of palm wine consumed (l), leaf dip rate (dips per min, calculated from video clips), estimated amount ethanol ingested (ml), and initials of researcher who contributed data.

| **Date** | **Session**  **no.** | **Event**  **no.** | **Start time** | **Chimp** | **Age** | **Sex** | **Dur.**  **(mins)** | **Palmwine**  **(l)** | **Dip rate**  **(dips/min)** | **Est. ethanol**  **(ml)** | **Researcher** |
| --- | --- | --- | --- | --- | --- | --- | --- | --- | --- | --- | --- |
| 08/07/1995 | 1 | 1 | 09:56:40 | Pi | 8 | F | 1.05 |  |  |  | GY |
| 28/12/1996 | 2 | 2 | 09:00:00 | FF | 16 | M | 7.52 | 0.70 | 7.30 | 18.8 | MN (Fig.2) |
|  |  | 3 |  | VUI | 10 | M | 4.45 |  | 11.01 |  |  |
|  |  | 4 |  | Jr | 38 | F | 2.50 | 0.23 | 12.00 | 6.3 |  |
| 01/08/2000 | 3 | 5 | 10:42:00 | TA | 43 | M | 21.00 | 1.94 |  | 52.5 | TH |
|  |  | 6 |  | FF | 19 | M | 12.00 | 1.11 |  | 30.0 |  |
|  |  | 7 |  | Pm | 33 | F | 3.00 | 0.28 |  | 7.5 |  |
| 17/07/2001 | 4 | 8 | 11:12:00 | FF | 20 | M | 32.00 | 2.96 |  | 79.9 | TH |
|  |  | 9 |  | Jr | 43 | F | 4.00 | 0.37 |  | 10.0 |  |
| 22/07/2001 | 5 | 10 | 14:02:00 | FF | 21 | M |  |  |  |  | GO |
|  |  | 11 |  | YL | 10 | M |  |  | 13.30 |  |  |
|  |  | 12 |  | Yo | Adult | F |  |  | 9.17 |  |  |
|  |  | 13 |  | Ju | 7 | F |  |  | 4.80 |  |  |
|  |  | 14 |  | Vl | Adult | F |  |  |  |  |  |
|  |  | 15 |  | JJ | 3 | M |  |  |  |  |  |
|  |  | 16 |  | Ka | Adult | F |  |  |  |  |  |
|  |  | 17 |  | Jr | 43 | F |  |  |  |  |  |
| 21/02/2003 | 6 | 18 | 11:32:00 | Pm | 36 | F | 1.00 | 0.09 |  | 2.5 | GO |
| 09/03/2003 | 7 | 19 | 07:00:54 | FF | 22 | M | 3.95 | 0.37 |  | 12.4 | GO |
| 05/02/2004 | 8 | 20 | 14:23:00 | YL | 12 | M | 17.00 | 1.57 |  | 53.5 | TH |
| 12/07/2004 | 9 | 21 | 14:13:31 | TA | 47 | M | 12.82 | 1.19 |  | 40.3 | GO |
|  |  | 22 |  | Pm | 37 | F | 23.00 | 2.13 |  | 72.3 |  |
|  |  | 23 |  | PE | 6 | M | 24.00 |  |  |  |  |
| 17/07/2004 | 10 | 24 | 17:54:39 | FF | 23 | M | 5.33 | 0.49 |  | 16.8 | GO.KH |
|  |  | 25 |  | YL | 13 | M | 6.00 | 0.56 |  | 18.9 |  |
|  |  | 26 |  | PE | 6 | M | 6.00 |  |  |  |  |
|  |  | 27 |  | JJ | 6 | M | 6.00 |  |  |  |  |
| 08/08/2004 | 11 | 28 | 12:18:49 | FF | 23 | M | 11.78 | 1.09 |  | 29.4 | GO |
|  |  | 29 |  | Pm | 37 | F |  |  |  |  |  |
|  |  | 30 |  | PE | 6 | M |  |  |  |  |  |
| 08/08/2004 | 12 | 31 | 15:30:28 | FF | 23 | M | 5.85 | 0.54 |  | 18.4 | GO |
|  |  | 32 |  | Pm | 37 | F |  |  |  |  |  |
|  |  | 33 |  | PE | 6 | M |  |  |  |  |  |
| 09/08/2004 | 13 | 34 | 13:16:54 | FF | 23 | M | 15.93 | 1.47 |  | 39.8 | GO |
|  |  | 35 |  | Pm | 37 | F |  |  |  |  |  |
|  |  | 36 |  | PE | 6 | M |  |  |  |  |  |
| 10/08/2004 | 14 | 37 | 08:11:00 | FF | 23 | M | 27.00 | 2.50 |  | 84.9 | KH |
|  |  | 38 |  | Pm | 37 | F | 27.00 | 2.50 |  | 84.9 |  |
|  |  | 39 |  | PE | 6 | M |  |  |  |  |  |
| 13/02/2005 | 15 | 40 | 11:28:00 | FF | 24 | M | 21.00 | 1.94 |  | 52.5 | KH |
|  |  | 41 |  | Jr | 47 | F | 4.00 | 0.37 |  | 10.0 |  |
|  |  | 42 |  | JJ | 7 | M | 4.00 |  |  |  |  |
| 2008 | 16 | 43 |  | FF | 28 | M |  |  | 10.70 |  | MF |
| 10/01/2009 | 17 | 44 | 14:53:31 | TA | 52 | M |  |  |  |  | SC |
|  |  | 45 |  | YL | 17 | M | 0.83 | 0.08 | 7.50 | 2.6 |  |
| 14/10/2011 | 18 | 46 | 12:05:00 | JJ | 13 | M | 1.00 | 0.09 | 5.26 | 2.5 | KH |
|  |  | 47 |  | Jr | 53 | F | 2.85 | 0.26 | 11.43 | 7.1 |  |
|  |  | 48 |  | FF | 31 | M | 2.48 | 0.23 | 9.26 | 6.2 |  |
| 22/12/2011 | 19 | 49 | 17:32:50 | FF | 31 | M | 3.52 | 0.33 | 10.59 | 11.1 | YY |
| 15/06/2012 | 20 | 50 | 09:16:49 | TA | 55 | M | 11.30 | 1.05 |  | 28.2 | NBM |
|  |  | 51 |  | JJ | 14 | M | 11.30 | 1.05 |  | 28.2 |  |

Video S1. Video clip of an adult male chimpanzee, Foaf, drinking fermented palm sap using a leaf tool.
